# Adverse drug reactions experienced by out-patients taking chlorpromazine or haloperidol at Zomba Mental Hospital, Malawi

**DOI:** 10.1186/s13104-019-4398-6

**Published:** 2019-07-01

**Authors:** Ibrahim Chikowe, McDonald Domingo, Vasco Mwakaswaya, Shagufta Parveen, Chitsanzo Mafuta, Elizabeth Kampira

**Affiliations:** 1 Pharmacy Department, College of Medicine, University of Malawi, Blantyre, Malawi; 2 Department of Pharmacology, Government Medical College Srinagar, Srinagar, India; 3 Zomba Mental Hospital, P.O. Box 38, Zomba, Malawi; 4 Medical Laboratory Sciences, College of Medicine, University of Malawi, Blantyre, Malawi

**Keywords:** Adverse drug reactions, Antipsychotic, Chlorpromazine, Haloperidol, Malawi, Mental health

## Abstract

**Objective:**

Drugs for managing mental disorders can cause adverse drug reactions (ADRs) that have negative impacts on patients yet, in Malawi, epidemiological data on the drug-related problems are limited. This study assessed the prevalence and severity of ADRs in out-patients at Zomba Mental Hospital.

**Results:**

Twenty-six of forty patients (65.0%) were taking haloperidol and 14 (35.0%) chlorpromazine. The commonest diagnosis was schizophrenia (n = 23, 57.5%) followed by epileptic psychosis (n = 4, 10.0%) and general psychosis (n = 4, 10.0%) with one of psychotic depression and one psychosis secondary to general medical condition. Comorbidities were also found with epilepsy being the commonest (n = 4, 10.0%). All patients reported at least one ADR of varying severity (mild, moderate and severe). Polydipsia was the most prevalent (24, 60.0%) followed by weight gain (20, 50.0%), spasm (15, 37.5%) and xerostomia (15, 37.5%). Some ADRs were gender specific and these included impotence (6/27, 29.6%) for males and menstrual changes (3/14, 21.4%) for females. Severe ADRs were more common in the older aged group (> 35 years 8.3% vs 7.1%), in males (11.1% vs 0.0%) and on chlorpromazine (14.3% vs 3.8%). Patients taking chlorpromazine and haloperidol are at risk of experiencing a wide range of ADRs with varying degrees of severity.

**Electronic supplementary material:**

The online version of this article (10.1186/s13104-019-4398-6) contains supplementary material, which is available to authorized users.

## Introduction

Mental disorders are one of the leading causes of ill-health and disability and almost one in four people are affected at some point in their life [1, 2]. In 2001, the World Health Organization (WHO) reported that 450 million people suffer from mental disorders which are becoming more serious in African countries due to limited healthcare services and an increase in population reaching the vulnerable ages [3]. Vulnerable age is the period from adolescence (10–14) when people experience many problems and practices that lead to mental disorders that have a high potential of developing into mental diseases. The predisposing factors include emotional disorders, mental health conditions, childhood behavioral disorders, eating disorders, psychosis, suicide and harm and risk-taking behavior [3]. In Malawi, studies have reported a high prevalence of probable common mental disorders ranging between 20 and 28% among primary health care patients [4, 5].

Antipsychotics are a class of medicines used to reduce psychotic symptoms. Although many antipsychotics are available and there has been an increase in their use, there has also been an increase in concerns about adverse drug reactions (ADRs), which have negative impacts on patient’s life, family lifestyle, economic losses [6, 7], therapy non-adherence and/or discontinuation [8]. Studies have been conducted worldwide to investigate early detection and management of ADRs [9–18]. In Malawi, there are limited epidemiological data on mental and drug-related health problems [19]. This study assessed the prevalence, severity and impact of ADRs on out-patients taking haloperidol or chlorpromazine at Zomba Mental Hospital.

## Main text

### Methods

#### Study design and setting

This was a cross sectional observational study conducted in the outpatient department at Zomba Mental Hospital in Malawi.

#### Data collection

The study targeted mentally stable adult out-patients more than 18 years old attending outpatient services, diagnosed with psychotic disorders and taking chlorpromazine or haloperidol for a single psychiatric episode. The qualified respondents were given the informed consent form to read, or have it read out to them. Only those that consented and signed or thumb-printed the consent forms were recruited. The study targeted all patients over a 4 weeks period. Clinicians and nurses at the hospital helped with identifying participants who met the enrolment criteria.

A structured questionnaire adapted from Katayi [20] was used. Patients were asked whether they wanted self-administration or to have it read out to them. Information regarding psychiatric diagnosis, other co-morbidities and medication history was obtained from patient health passports and medical files. Demographic information, patient clinical data, prescribed medicines and side effects were entered in Microsoft Excel (2013) for analysis using descriptive statistics according to drug, gender, age, and organ system. Severity of ADRs was analyzed according to Glasgow antipsychotic side effects scale (GASS). This is a 22-item self-reporting questionnaire for identification of ADRs caused by antipsychotics [21].

### Results

#### Characteristics of the study participants

Forty-three psychotic participants qualified for the study and 40 participated (93.0% acceptance). The participants were taking at least one antipsychotic drug (chlorpromazine or haloperidol). 67.5% (n = 27) were males. Mean age was 31.4 years; range 18–57 years. 50.0% (20/40) of the participants reached at least secondary school level, while 5.0% (2/40) reached college level. Forty-five percent (18/40) of the participants never married, and 40.0% (16/40) were unemployed.

Over half (57.5%, n = 23) were diagnosed with schizophrenia, while the rest included epileptic psychosis (10.0%, n = 4), general psychosis (10.0%, n = 4), bipolar affective disorder (7.5%, n = 3), schizoaffective disorder (5.0%, n = 2), cannabis-induced psychosis (5.0%, n = 2), psychotic depression (2.5%, n = 1) and psychosis secondary to general medical condition (2.5%, n = 1). Some patients presented with co-morbid physical illnesses including epilepsy (10.0%, 4/40), HIV/AIDs (7.5%, 3/40), and hypertension (5.0%, 2/40). Five percent (n = 2) presented with depression as a secondary diagnosis. Additional file 1: Table S1 outlines study participants details.

#### Reasons for prescribing the reported medicines

Two-thirds of participants (65%; n = 26) were taking haloperidol while 35% (n = 14) took chlorpromazine; 55% (n = 22) took only one antipsychotic drug but 45% (n = 18) had an adjunct treatment. Benzhexol was used in 4 patients (10%) to control extrapyramidal side-effects. Co-morbidities were managed by a wide range of medicines like carbamazepine (10.0%, n = 4), fluoxetine, sodium valproate, hydrochlorothiazide, amitriptyline (one each) and antiretroviral therapy (ART) (7.5%, n = 3).

#### Prevalence of ADRs

All participants reported at least one ADR. Polydipsia with polyuria was the most prevalent (60.0%, n = 24) followed by weight gain (50.0%, n = 20), sedation (47.7%, n = 19), arrhythmia (42.5%, n = 17), spasm (37.5%, n = 15), xerostomia (37.5%, n = 15), tremor (35.0%, n = 14), restlessness (32.5%, n = 13), daze (30.0%, n = 12), dizziness (30.0%, n = 12), bradykinesia (30.0%, n = 12), malaise (27.5%, n = 11), constipation (27.5%, n = 11), blurred vision (22.5%, n = 9), tardive dyskinesia (20.0%, n = 8), salivation (12.5%, n = 5), difficulty in urination (12.5%, n = 5), sore nipple (10.0%, n = 4), nipple discharge (7.5%, n = 3) and bed wetting (7.5%, n = 3) (Additional file 2: Table S2).

22.2% of men (6/27) experienced impotence and 30.8% (4/13) women experienced menstrual changes. 29.0% (9/31) of those who admitted to be sexually active had reduced orgasm during sexual intercourse.

#### Occurrence of ADRs based on gender and drug regimen

**Fig. 1:**
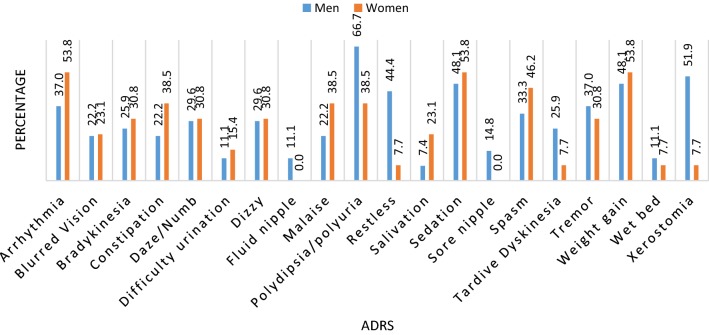
Prevalence of ADRs in male (men) and female (women) respondents

**Fig. 2:**
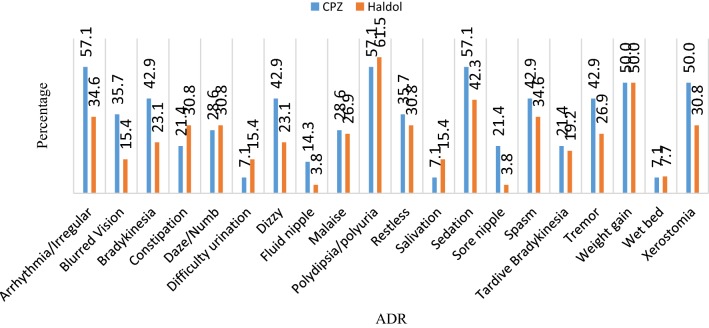
Prevalence of ADRs caused by chlorpromazine (CPZ) and haloperidol (Haldol)

The ADRs were also analyzed on the basis of gender. Polydipsia had the highest occurrence in men (66.7%, n = 18), while sedation (53.8%, n = 7), weight gain (53.8%, n = 7) and arrhythmia (53.8%, n = 7) were the highest occurring ADRs in women (Fig. 1). Of the patients taking haloperidol, 61.5% (16/26) reported polydipsia, while patients taking chlorpromazine reported arrhythmia (57.1%, n = 8), polydipsia (57.1%, n = 8) and sedation (57.1, n = 8) (Fig. 2).

#### Severity of the ADRs

Adverse drug reactions were analyzed for severity using GASS criteria. A severe reaction is one that needs intensive medical care or requires advanced treatment procedures, may cause permanent harm or lead to death directly or indirectly, and requires greater financial expenditure from the patients. 7.5% (3/40) of all respondents experienced severe ADRs; 2/14 to chlorpromazine and 1/26 to haloperidol (see Table 1).

**Table 1:** ADR severity according to age, gender and drug

Parameter	Variable	GASS severity					
		Absent/mild		Moderate		Severe	
		N	%	N	%	N	%
Age	Young adult; 18–35 years (N = 28)	22	78.6	4	14.3	2	7.1
	Middle aged adult; > 35 years (N = 12)	6	50.0	5	41.7	1	8.3
Gender	Male (N = 27)	19	70.3	5	18.5	3	11.1
	Female (N = 13)	9	69.2	4	30.8	0	0.0
Drug	Haloperidol (N = 26)	22	84.6	3	11.5	1	3.8
	Chlorpromazine (N = 14)	6	42.9	6	42.9	2	14.3

### Discussion

Typical (first generation) antipsychotic drugs (chlorpromazine and haloperidol) are still the first line prescribed antipsychotic medications at Zomba Mental Hospital and this study has shown that every patient experienced adverse drug reactions. This is similar to management of outpatients at Mathari hospital in Kenya [20], Butabika National Referral Mental Hospital in Uganda [22] and Amanuel Mental Specialized Hospital in central Ethiopia [23]. However, it was in contrast with a psychiatry hospital in Kashmir, India where atypical antipsychotic drugs were the first line antipsychotic medications [10]. In the USA, a survey in 2004–2005 found that three-quarters of patients took SGAs [24]. In our study, the three most common ADRs for both medications were polydipsia, weight gain and sedation. Significant number of patients had co-morbidities including HIV/AIDS, which require the psychiatrist’s careful evaluation of treatment options due to their side effects and drug interactions [25, 26].

Male predominance for some ADRs like polydipsia (excessive thirst) and/or polyuria have been reported (Figs. 1, 2). Munoli and Patil [27], Afkat et al. [9] and Katayi [19] also reported a male predominance for those ADRs. However, Munoli and Patil [27] and Katayi [20] reported sedation as the most observed ADR mainly contributed by chlorpromazine. Polydipsia was found for both chlorpromazine and haloperidol and may result in life threatening hyponatremia [28–31]. Adjunct therapy with the use of drugs like tolvaptan has been shown to improve hyponatremia but is expensive in Africa [30, 32].

Weight gain and sedation were second and third most commonly experienced ADRs respectively (Figs. 1, 2). Sedation caused mainly by chlorpromazine was also the most common male ADR reported in Guntur, Andhra Pradesh [33], but in the current study, sedation was also the most common ADR experienced among the female population (Figs. 1, 2). Sedation induced by antipsychotics has been shown to improve with adjunct medication like modafinil [34], which not only helps patients feel alert, but also prevents sedation-induced weight gain [35]. Other antipsychotic drugs like aripiprazole lack obesity-promoting pharmacology [35].

Sexual dysfunction effects (impotence, reduced orgasm, premature ejaculation) of antipsychotics, although experienced by few participants, posed a negative impact on patients’ social behavior and quality of life. Park et al. [36] reviewed evidence from a large prospective study of sexual dysfunctions that showed that similar ADRs were experienced by 71.1% of men and women taking typical antipsychotics. Antipsychotic-induced sexual dysfunction improved with the use of adjunct drugs like sildenafil, bromocriptine, amantadine and cabergoline [37, 38].

Adverse drug reactions that posed a negative impact on patient’s social life included sedation, reduced orgasm, impotence and premature ejaculation (Additional file 2: Table S2). Affected participants complained that the sedative effects made them passive at their work. Some participants reported to have divorced or left their marriage partners due to sexual dissatisfaction. Although polydipsia and weight gain were amongst three major ADRs commonly experienced by patient participants, they were tolerable and manageable. Other studies have also found that the ADRs have a negative impact on patients physical appearance and social life [10, 12, 25].

Roughly the same proportion of respondents reported severe ADRs (7.5%; 3/40) as in the USA (9%). More severe cases in this study appeared to be in patients taking chlorpromazine as opposed to haloperidol in a USA study [16].

Both FGAs and SGAs are in use in different parts of the world, chosen according to treatment outcomes and adverse effects. Both groups have significant ADRs though they are different. While the FGAs’ adverse effects are strongly associated with dry mouth, sedation, extrapyramidal symptoms, and tardive dyskinesia, some of which are irreversible, SGAs were developed to overcome the difficulty of dealing with those adverse effects. However, they also have their own side effects such as dyslipidaemia, weight gain, metabolic syndrome and diabetes mellitus. In both cases, the adverse effects are capable of influencing non-adherence to treatment regimen and poor treatment outcome. Other studies have shown no significant results in comparisons between FGAs and SGAs effectiveness and adverse effects profiles of drugs from the two groups but individual patients may respond quite differently to any two drugs. As such, clinical decision making depends on thorough clinical evaluation of data and individual characteristics [24].

Further studies are needed in Malawi to find if there might be a need to switch to the SGAs or remain with the FGAs. Murphy and McMahon [39] reported that treatment outcomes vary with racial group and ethnicity due to genetic polymorphism that leads to pharmacokinetic and pharmacodynamic variations as well as genotype–phenotype variations. Further studies are needed on a variety of local races and ethnic groups on the presently used FGAs [39]. This is crucial in antipsychotic treatment regimens as ADRs have long-term effects on adherence and achievement of successful treatment [10, 40].

### Conclusion

Patients taking chlorpromazine or haloperidol may be experiencing different ADRs. A high prevalence of ADRs due to treatment with chlorpromazine or haloperidol has imposed a significant burden on the patients. These may have negative impact on the patient’s sustenance of the treatment regimen. Therefore efforts should be made to prevent or mitigate the common ADRs of weight-gain, sedation and sexual disturbances. Routine use of second generation antipsychotics should be considered.

## Limitation of the study

The study targeted a small number of patients within a short period of time; hence the results cannot be generalized. However, this can act as a signal that may help in the design of a more inclusive study that can target larger populations.

## Additional files

**Additional file 1: Table S1.** Demographic and health information about respondents.

**Additional file 2: Table S2.** ADRs from specified population and others.

## Data Availability

The datasets used and/or analyzed during the current study are available from the corresponding author on reasonable request.

## Abbreviations

ADR: adverse drug reaction
ART: anti-retroviral therapy
BPAD: bipolar affective disorder
GASS: Glasgow antipsychotic side effects scale
HIV: human immunodeficiency virus
