# Supplementary material for: Adverse drug reactions experienced by out-patients taking chlorpromazine or haloperidol at Zomba Mental Hospital, Malawi
Source: BMC Res Notes. 2019 Jul 1;12:376. doi: 10.1186/s13104-019-4398-6 (PMC6604158; doi:10.1186/s13104-019-4398-6)
Supplement: Supplementary file 1 — Additional file 1: Table S1. Demographic and health information about respondents. [file 13104_2019_4398_MOESM1_ESM.docx]

| **123Class** | **Variable** | **Count (N=40)** | **Percentage (%)** |
| --- | --- | --- | --- |
| Age (years) | 18-20 | 2 | 5.0 |
|  | 21-30 | 17 | 42.5 |
|  | 31-40 | 15 | 37.5 |
|  | 41-50 | 4 | 10.5 |
|  | 51-60 | 2 | 5.0 |
| Gender | Male | 27 | 67.5 |
|  | Female | 13 | 32.5 |
| Education | Informal | 4 | 10.0 |
|  | Primary | 14 | 35.0 |
|  | Secondary | 20 | 50.0 |
|  | College/University | 2 | 5.0 |
| Marital status | Single | 18 | 45.0 |
|  | Married | 12 | 30.0 |
|  | Separated | 1 | 2.5 |
|  | Divorced | 8 | 20.0 |
|  | Widowed | 1 | 2.5 |
| Employment | Student | 6 | 15.0 |
|  | Unemployed | 16 | 40.0 |
|  | Employed | 5 | 12.5 |
|  | Self-employed | 13 | 32.5 |
| Psychiatric Diagnosis | Schizophrenia | 23 | 57.5 |
|  | Epileptic psychosis | 4 | 10.0 |
|  | General psychosis | 4 | 10.0 |
|  | BPAD | 3 | 7.5 |
|  | Schizoaffective disorder | 2 | 5.0 |
|  | Cannabis psychosis | 2 | 5.0 |
|  | Psychotic depression | 1 | 2.5 |
|  | Psychosis secondary to general medical condition | 1 | 2.5 |
| Co-morbidities | Hypertension | 2 | 5.0 |
|  | Depression | 2 | 5.0 |
|  | HIV Reactive | 3 | 7.5 |
|  | Epilepsy | 4 | 10.0 |
| Antipsychotics | Haloperidol | 26 | 65.0 |
|  | Chlorpromazine | 14 | 35.0 |
| Adjuncts (medications for co-morbidities or ADRs) | Benzhexol | 4 | 10.0 |
|  | Carbamazepine | 4 | 10.0 |
|  | Fluoxetine | 1 | 2.5 |
|  | Sodium valproate | 1 | 2.5 |
|  | Hydrochlorothiazide | 1 | 2.5 |
|  | Amitriptyline | 1 | 2.5 |
|  | 6A ART Regimen | 1 | 2.5 |
|  | 2A ART Regimen | 1 | 2.5 |
|  | 5A ART Regimen | 1 | 2.5 |

^BPAD = bipolar affective disorder HIV = human immunodeficiency virus ART = antiretroviral therapy^
