# Supplementary material for: Adverse drug reactions experienced by out-patients taking chlorpromazine or haloperidol at Zomba Mental Hospital, Malawi
Source: BMC Res Notes. 2019 Jul 1;12:376. doi: 10.1186/s13104-019-4398-6 (PMC6604158; doi:10.1186/s13104-019-4398-6)
Supplement: Supplementary file 2 — Additional file 2: Table S2. ADRs from specified population and others. [file 13104_2019_4398_MOESM2_ESM.docx]

| **Class** | **Body system(s)** | **Variable** | **Count (n/N)** | **Percentage (%)** |
| --- | --- | --- | --- | --- |
| ADRs from specified population | - Psychiatric - Sexual and reproductive | Less orgasm | 9/31 | 29.0 |
|  |  | Impotence | 6/27 | 22.2 |
|  |  | Premature ejaculation | 1/27 | 3.7 |
|  |  | Menstrual changes | 4/13 | 30.8 |
| ADRs experienced by participating patients | - Metabolic and nutritional | Polydipsia/polyuria | 24/40 | 60.0 |
|  |  | Weight gain | 20/40 | 50.0 |
|  | - Psychiatric - Central and peripheral nervous | Sedation | 19/40 | 47.5 |
|  |  | Frequent yawns | 1/40 | 2.5 |
|  | - Cardiovascular | Arrhythmia | 17/40 | 42.5 |
|  | - Central and peripheral nervous | Spasm | 15/40 | 37.5 |
|  |  | Tremor | 14/40 | 35.0 |
|  |  | Daze/numbness | 12/40 | 30.0 |
|  |  | Dizziness | 12/40 | 30.0 |
|  |  | Bradykinesia | 12/40 | 30.0 |
|  |  | Tiredness/Weakness | 3/40 | 7.5 |
|  |  | Severe sweating | 1/40 | 2.5 |
|  |  | Tardive dyskinesia | 8/40 | 20.0 |
|  |  | Neck stiffness | 2/40 | 5.0 |
|  | - Gastrointestinal - Digestive | Xerostomia | 15/40 | 37.5 |
|  |  | Constipation | 11/40 | 27.5 |
|  |  | Salivation | 5/40 | 12.5 |
|  |  | Severe hunger | 2/40 | 5.0 |
|  |  | Diarrhea | 1/40 | 2.5 |
|  | - Neurological - Psychiatric | Restlessness | 13/40 | 32.5 |
|  | - Psychiatric - Neurological | Malaise | 11/40 | 27.5 |
|  |  | Depression | 2/40 | 5.0 |
|  | - Central and peripheral - Cardiovascular | Blurred vision, | 9/40 | 22.5 |
|  | - Urinary | Difficulty in Urination | 5/40 | 12.5 |
|  |  | Bed Wetting | 3/40 | 7.5 |
|  | - Sexual and reproductive | Sore nipple | 4/40 | 10.0 |
|  | - Lymphatic | Nipple discharge | 3/40 | 7.5 |
|  | - Psychiatric | Short temper | 1/40 | 2.5 |
|  |  | Insomnia | 2/40 | 5.0 |
|  | - Neurological | Short term memory loss | 2/40 | 5.0 |
|  | - Immune | Sore throat | 1/40 | 2.5 |
